# Multifaceted leaders: the double-edged sword effect of narcissistic leadership on employees’ work behavior

**DOI:** 10.3389/fpsyg.2023.1266998

**Published:** 2024-01-24

**Authors:** Hao Chen, Lei Zhang, Liang Wang, Jiaying Bao, Zihan Zhang

**Affiliations:** ^1^School of Public Health and Management, Youjiang Medical University for Nationalities, Baise, Guangxi, China; ^2^Clinical Research Center for Mental Disorders, Shanghai Pudong New Area Mental Health Center, School of Medicine, Tongji University, Shanghai, China; ^3^School of Economics and Management, Wuxi Vocational Institute of Arts and Technology, Wuxi, Jiangsu, China; ^4^School of Languages and Cultures, Youjiang Medical University for Nationalities, Baise, Guangxi, China; ^5^School of Economics and Management, Wuhan University, Wuhan, Hubei, China

**Keywords:** narcissistic leadership, hostility toward supervisor, psychological availability, counterproductive work behavior, organizational citizenship behavior, environment uncertainty

## Abstract

So far, most studies have focused on exploring the negative effects of narcissistic leadership. However, little attention has been paid to whether narcissistic leadership also has a positive effect. This study is based on Conservation of Resources Theory and reveals that narcissistic leadership has a double-edged sword effect. By using Mplus7.4 software the analysis of 450 employees and their direct leaders’ pairing data collected in three stages, it is found that: narcissistic leadership has a positive effect on employee’ hostility toward supervisor and psychological availability; hostility toward supervisor mediates the relationship between narcissistic leadership and counterproductive work behavior; psychological availability mediates the relationship between narcissistic leadership and organizational citizenship behavior; in addition, environmental uncertainty enhances the positive effect of narcissistic leadership on employee’ hostility toward supervisor and psychological availability, which in turn moderates the indirect effect of narcissistic leadership on counterproductive work behavior through employee’ hostility toward supervisor and on organizational citizenship behavior through psychological availability.

## Introduction

Leadership has long been a hot topic in academic circles with many scholars focusing their research on the “glossy sides” of leadership (Zhang et al., 2017; Javed et al., 2019). In recent years, the “dark side” of leadership has been gaining attention from scholars (Ateş et al., 2020; Chen H. et al., 2023; Chen C. et al., 2023). Among them, narcissistic leadership with narcissistic traits in dark personalities is increasingly attracting the attention of scholars (Braun et al., 2018; Carnevale et al., 2018). However, celebrities with typical narcissistic leadership traits, such as Steve Jobs, Bill Gates, and Abraham Lincoln, had or have indeed achieved remarkable success. As a result, some scholars have also begun to turn their research lens to the positive mechanisms of narcissistic leadership (Chatterjee and Hambrick, 2007; Harms et al., 2011).

At present, although research on narcissistic leadership has made some progress, the empirical results mainly focus on the negative effects of narcissistic leadership (Nevicka et al., 2011a; Badar et al., 2023). Although some studies have provided important clues for the positive effects of narcissistic leadership (Owens et al., 2015; Wang et al., 2022), their single research perspective only produces a one-sided understanding of narcissistic leadership, lacking further confirmation from other perspectives that narcissistic leadership has a positive effect. In light of this, this paper intends to comprehensively reveal the double-edged sword effect of narcissistic leadership from a dialectical perspective.

With the development of organizational behavior research, more and more studies have gradually shifted from focusing on employees’ task performance to employees’ extra-role behaviors. Among them, counterproductive work behaviors and organizational citizenship behaviors are two very common and highly relevant extra-role behaviors in management practice, the former focusing on spontaneous behaviors that deplete the organization or members’ interests (Mitchell et al., 2022), and the latter focusing on spontaneous or mutually supportive behaviors that enhance organizational performance (Qiu and Dooley, 2022). Research has shown that leadership behaviors are important antecedents that influence employees’ behavioral decisions (Carter and Baghurst, 2014). Following this logic, are employees’ counterproductive work behaviors and organizational citizenship behavior also influenced by narcissistic leadership? If so, what mediation mechanism is the impact transmitted through? In what context does this mediation mechanism exhibit differences? However, few scholars have explored this aspect of research.

To answer the above questions, first, based on Conservation of Resources Theory, we construct a logical framework between narcissistic leadership and employee work behavior by taking the depletion and supplementation of individual resources as the starting point. Second, we choose two important variables that may have effects between leadership behavior and employee behavior, they are hostility toward supervisor and psychological availability. Hostility toward supervisor indicates an individual’s hostile emotional state toward supervisor (Du et al., 2017), and psychological availability indicates an individual’s perception of the resources available to accomplish work (Li and Tong, 2021), both of which are closely related to the stock of resources available to the individual. Thus, we intend to explore mechanism of action between narcissistic leadership and counterproductive work behavior as well as organizational citizenship behavior, through two pathways of hostility toward supervisor and psychological availability. In addition, environmental uncertainty, as the mainstream external environment today, plays an important role in influencing the performance of leadership behavior (Zhang, 2021). Therefore, environmental uncertainty may be an important boundary condition in the process of narcissistic leadership influencing hostility toward supervisor and psychological availability.

**Figure 1:**
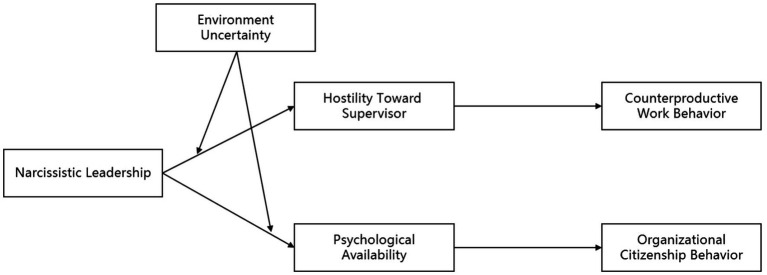
The theoretical model.

In summary, this study takes the dual nature of narcissistic leadership as the starting point, deeply analyzes the impact mechanism and role boundaries of narcissistic leadership on employee counterproductive work behavior and organizational citizenship behavior, reveals the mechanism behind the double-edged sword effect of narcissistic leadership, and promotes the continuous development of the research field of narcissistic leadership through empirical research. Meanwhile, this study provides useful practical guidance for effectively inhibiting the negative side of narcissistic leadership, stimulates the positive side of narcissistic leadership, reduces employee counter productive behaviors, and promotes the generation and development of organizational citizenship behaviors among employees. The specific research model is shown in Figure 1.

## Literature review and hypotheses

### Conservation of resources theory

Conservation of Resources Theory suggests that individuals have a limited amount of resources and they have a tendency to acquire, maintain and protect resources, and any loss or depletion of resources is a threat to the individual (Hobfoll, 1989). Instead of suffering easily from resource loss, individuals with sufficient resources are more capable of acquiring them, and the new resources they acquire create greater resource increments, thus entering the value-added spiral. However, resource acquisition spirals do not form as quickly as loss spirals, so individuals who lack resources are more likely to fall into loss spirals (Hobfoll, 1989). Based on the analyses above, we infer that narcissistic leaders influence their behavioral choices by depleting or supplementing employee resources.

### Narcissistic leadership and hostility toward supervisor, psychological availability

Narcissism is a personality trait that encompasses arrogance, hubris, self-focus, fragile self-esteem, and hostility, which is present in many strong leaders (Yao et al., 2019; Li and Tong, 2021). Narcissistic leaders are those who are influenced by the narcissistic personality and whose behaviors are driven by self-interested needs and beliefs, with less concern for the interests of the subordinates they lead and the organization (Rosenthal and Pittinsky, 2006). Most scholars consider narcissistic leaders as a more negative type of leaders (Schyns et al., 2023) and categorize them as egoists who use all available resources to help themselves gain the respect of others and as a way to gain superiority over themselves (Resick et al., 2009). However, it has also been suggested that narcissistic leaders have a positive effect, with better self-insight (Grijalva and Harms, 2014; Ghislieri et al., 2019), possessing a strong leadership charisma and the ability to achieve a grand vision (O'Reilly et al., 2014). Therefore, narcissistic leadership’ glossy and dark sides may bring different mechanisms of influence on employees’ emotions, cognition, and behavior.

Hostility toward supervisor as a negative emotion is an emotional state that tends to be a hostile, defiant, or even aggressive willingness towards others when individuals’ desire to be valued or respected is not realized, or when they feel they are rejected, insulted, treated unfairly, or experience frustration (Du et al., 2017). According to Conservation of Resources Theory, negative leadership behavior can lead to employees being unable to obtain effective resource compensation, thereby damaging their psychological and cognitive resources (Morrison and Milliken, 2000). Therefore, this paper argues that narcissistic leadership triggers employee hostility toward supervisor.

Specifically, narcissistic leaders have the tendency to be selfish and exploitative of others, and will exhibit more negative behaviors towards subordinates at work (Tokarev et al., 2017). For example, stealing and appropriating subordinates’ work results, denigrating subordinates, and being overly harsh to subordinates (Chen et al., 2018). These behaviors seriously threaten and deplete employees’ own resources, and due to the lack of sufficient resources for effective emotional and cognitive management (Bacharach and Bamberger, 2007), employees feel hurt and belittled, which will lead to strong hostility towards leaders (Lazarus, 1991). In addition, narcissistic leaders have a strong sense of superiority and control, lack empathy, and have a tendency to exploit others, which leads to a lack of autonomy and difficulty in feeling support from the leaders (Xiao et al., 2018), resulting in employees’ inability to obtain new resources to replenish, which leads to hostility toward supervisor. Therefore, the following hypothesis is proposed.

*H1:* Narcissistic leadership has a positive effect on employee’ hostility toward supervisor.

Psychological availability is a perception of an individual’s availability of his or her physical, emotional, and cognitive resources at a given moment (Kahn, 1990) and reflects the degree to which an individual perceives that he or she can cope with the physical, emotional, and cognitive demands of the job (Zhang et al., 2023). Among them, physiological needs are the most basic needs, which are the strength, endurance and flexibility needed to complete tasks; emotional needs are the mental states and emotions that affect employees’ activities; and cognitive needs are the knowledge and skills that employees learn to complete complex tasks (Cai et al., 2018). Psychological availability is essentially a psychological expectation and confidence that individuals have in the resources they receive at work, helping people to decide whether and how to engage in organizational activities (Li and Tong, 2021).

According to Conservation of Resources Theory, individuals can better accumulate resources when there is less pressure at work (Hobfoll, 2001), leaders, as an important situational element (Chen H. et al., 2023; Chen C. et al., 2023), can enable employees to store the resources they provide when interacting with them, thus enable employees to increase their own resources, and obtain critical psychological resources. Therefore, this study argues that narcissistic leadership can promote psychological availability. Firstly, narcissistic leaders usually have the traits of self-confidence, extroversion, humor, and have certain personal charisma and social skills (Li and Tong, 2021). With this trait, narcissistic leaders are able to make employees emotionally fulfilled, alleviates interpersonal conflicts and stress, and to some extent enhances employees’ confidence in showing appropriate emotions at work, which enhances employees’ perceived availability of emotional resources.

Secondly, narcissistic leaders are more aggressive, passionate, and possess a strong work ethic (Schyns et al., 2023), which can enhance employees’ perceived job stability and security, satisfies their basic needs to complete tasks, avoids resource depletion due to work environment stress and uncertainty, and greatly improves the perceived availability of physiological resources (Xu and Yang, 2023). In addition, narcissistic leaders have an ambitious vision and dare to innovate, which can motivate employees to continuously learn new skills, explore new knowledge, and improve their problem-solving skills, greatly improving the perceived availability of cognitive resources. Therefore, the following hypothesis is proposed.

*H2:* Narcissistic leadership has a positive effect on psychological availability.

### The mediation role of hostility toward supervisor and psychological availability

Counterproductive work behaviors are actions committed by employees in the workplace that intentionally harm organizational interests or other members associated with organizational interests (Bennett and Robinson, 2000) and include overt aggression and stealing, handling personal business during work hours, intentional failure to follow instructions and misassignments, and other more negative actions that are directly detrimental to the organization’s functions and assets (Mitchell et al., 2022). Conservation of Resources Theory suggests that individuals are motivated to protect or compensate for resources when faced with the threat of resource loss (Hobfoll, 1989). Thus, employees who develop hostile feelings are in a stressful state of resource loss, and they may engage in counterproductive behaviors in response to the stress to stop the continued depletion of resources and to make up for the lost resources.

On the one hand, employees who generate hostile emotions themselves lack the psychological resources to manage their emotions and behaviors (Tokarev et al., 2017), and it is inevitable that during their interactions with leaders, they may have difficulty regulating their negative emotions and verbally attack the leaders, or treat their own work negatively in negative emotions due to their limited energy (Xiao et al., 2018). On the other hand, hostility toward supervisor is often accompanied by negative emotions such as anger and disgust (Chen C. et al., 2023), and in order to alleviate these negative emotions caused by the loss of resources, employees may use their jobs for personal gain to satisfy their own interest needs, i.e., to obtain resources by stealing company assets, and other behaviors to alleviate the pressure of resource loss (Berkowitz, 1989). Thus, hostile emotions consume a large amount of individual resources and easily induce employees to respond with counterproductive work behaviors. Therefore, the following hypothesis is proposed.

*H3*: Hostility toward supervisor plays a mediation role in the relationship between narcissistic leadership and employee counterproductive work behavior.

Organizational citizenship behavior refers to those extra-role behaviors that employees untarily adopt in addition to formal job requirements (Lee and Allen, 2002), which are not included in the formal compensation system but help to build harmonious interpersonal relationships, improve organizational effectiveness, and enhance organizational cohesion and influence (Qiu and Dooley, 2022). Based on Conservation of Resources Theory, narcissistic leaders can provide employees with the resources they need, and employees whose needs are met can better help other employees. Specifically, when employees have higher perceptions of the physical, emotional, and cognitive resources needed to complete their work tasks, employees are able to have good physical fitness and physiological health (Cai et al., 2018) and are able to maintain friendly emotional connections with their leaders (Milad et al., 2017).

At the same time, employees with high psychological availability are clear-headed, have high confidence in their abilities (Gerdientje et al., 2013), are good at clarifying their thinking, proactive in learning and focused on their work (Russo et al., 2016). They are good at contributing their wisdom, they are more proactive to better accomplish their work goals, and they believe that their efforts will contribute to the organizational vision achievement, and even further generate more behaviors from within that benefit others and the organization. Therefore, the following hypothesis is proposed.

*H4:* Psychological availability mediates the relationship between narcissistic leadership and employee organizational citizenship behavior.

### The moderation role of environmental uncertainty

Environmental uncertainty is the perception that individuals cannot accurately predict the external environment of an organization, and it reflects the degree to which the external environment is unpredictable and unstable (Milliken, 1987). Conservation of Resources Theory suggests that individuals have limited resources (Hobfoll, 1989), when employees perceive a high level of environmental uncertainty, they may perceive the organization as at risk, generating more anxiety and stress (Waldman et al., 2001), which in turn exacerbates the depletion of their own resources. Therefore, in this situation, narcissistic leaders overestimate their own contributions while ignoring others’ contributions, suppressing others’ suggestions, and attributing the organization’s success to negative traits of themselves, which may exacerbate employees’ hostility and interpersonal conflicts towards narcissistic leaders (Siebens, 2019). In contrast, when employees perceive lower levels of environmental uncertainty, they experience less anxiety and stress (Zhang, 2021), and the narcissistic leadership behaviors exhibited by leaders in this context may not be disproportionately negatively impacted for employees, which in turn may not further exacerbate the impact on employee hostility toward supervisor. Therefore, the following hypothesis is proposed.

*H5:* Environmental uncertainty plays a moderation role between narcissistic leadership and hostility toward supervisor. That is, the higher the environmental uncertainty is, the stronger the positive relationship between narcissistic leadership and hostility toward supervisor is.

In addition, this study argues that the strength of the relationship between narcissistic leadership and psychological availability is influenced by environmental uncertainty. Specifically, when employees perceive higher levels of environmental uncertainty, narcissistic leaders have a grand sense of self-importance, higher confidence and charisma, and through the use of engaging expressions and highly contagious words, they are able to motivate employees to change their status quo and equip them with the confidence and ability to deal with problems, which, to a certain extent, alleviates the degree of internal stress and resource depletion of employees, thus increasing psychological availability (Zhang et al., 2023). When employees perceive a high level of environmental uncertainty, they may be less willing to consume resources to communicate with the leader, and they may think that the leader is only superficially fair and reasonable, but they will t really listen to him or her, and they cannot reach an agreement, thus reducing psychological availability (Milad et al., 2017). Therefore, the following hypothesis is proposed.

*H6:* Environmental uncertainty plays a moderation role between narcissistic leadership and psychological availability. That is, the higher the environmental uncertainty is, the stronger the positive relationship between narcissistic leadership and psychological availability is.

### Moderated mediation effects

Based on Conservation of Resources Theory and the hypotheses, it can be further inferred that the mediation effects of hostility toward supervisor and psychological availability may be influenced by environmental uncertainty. Specifically, under high environmental uncertainty, the relationship between the “dark side” of narcissistic leadership and employees’ hostility is stronger, and the increase in employees’ hostility is more likely to exacerbate counterproductive behaviors (Chen H. et al., 2023; Chen C. et al., 2023). The “bright side” of the narcissistic leadership has a stronger relationship with the psychological availability of employees and is more likely to promote organizational citizenship behavior.

Conversely, low environmental uncertainty reduces employees’ susceptibility to narcissistic leadership behaviors, thereby weakening the effect of narcissistic leadership on employee hostility, and less hostile employees exhibit less counterproductive behaviors. It also reduces the facilitative effect of narcissistic leadership on their psychological availability (Xu and Yang, 2023) and reduces the frequency of employees’ organizational citizenship behaviors. Therefore, the following hypotheses are proposed.

*H7*: Environmental uncertainty moderates the mediation effect of hostility toward supervisor between narcissistic leadership and employee counterproductive work behavior, i.e., the higher the environmental uncertainty is, the stronger the mediation effect of hostility toward supervisor between narcissistic leadership and employee counterproductive work behavior is.

*H8*: Environmental uncertainty moderates the mediation effect of psychological availability between narcissistic leadership and employee organizational citizenship behavior, i.e., the higher the environmental uncertainty is, the stronger the mediation effect of psychological availability between narcissistic leadership and employee organizational citizenship behavior is.

## Method

### Participants and procedure

This study mainly takes employees and their direct supervisors from several mature enterprises in China (including manufacturing, information technology, service, and finance industries) as samples, and collects data through offline questionnaire responses. In order to mitigate the effects of homologation bias, this study conducted a 1:1 employee-direct supervisor matching approach to data collection at three-time points, with an interval of 1 month. The survey process is as follows: at time point 1 (T1) the survey was administered to employees which included basic information about the employee and narcissistic leaders; at time point 2 (T2) the survey was administered to the employees which included hostility toward supervisor, psychological availability and environment uncertainty; at time point 3 (T3) the survey was administered to the employees’ direct supervisors which included employees’ counterproductive work behavior and organizational citizenship behavior. In order to avoid the influence of the “golden mean” thought in Chinese culture on the participants, that is, to overcome the “centricity effect” when filling in the items of the scale, except for some demographic variables, all questionnaires in this study use the Likert 6-point scoring method adopted by many scholars (Wei et al., 2023).

In order to enable participants to complete the questionnaire correctly, we took the following five specific measures during the data collection. First, with the cooperation of the human resources department of the enterprises, we obtained a list of research subjects, and coded them based on the organizational structure of each department from the list. Considering the working relationship and familiarity between employees and leaders, we selected paired individuals who work in the same department. We used a random sampling method to select one employee and one leader for pairing, and ensured that each research subject matched a different person. Second, before distributing the questionnaires, we explained to all participants that the data collected in the questionnaires was only for academic research, not for any other purpose. Third, we promised to pay 50 yuan (about $7) per person after completing three surveys correctly. Fourth, in the process of answering the questionnaire, one of our members maintained a close relationship with the participants to solve any problems they raised. Finally, after the participants completed the questionnaires, we checked the questionnaires to ensure that there was no missing data. Then, we immediately collected, sealed and encoded the questionnaires.

In the first survey, 485 employee questionnaires were distributed on-site, and 474 valid questionnaires were returned. In the second survey, targeted distribution was conducted to employees who provided valid questionnaires in the first survey and a total of 462 valid questionnaires were returned. In the third survey, targeted questionnaires were distributed to the direct supervisors of employees who provided valid questionnaires for the second time, and finally 450 valid matched questionnaires between employees and direct supervisors were obtained (return rate is 92.78%). In terms of sample structure, male employees predominate (59.1%), in terms of age structure, young people predominate, employees under the age of 35 (81.1%), in terms of education structure, junior college and above predominate (72.9%). The basic information of the samples is shown in Table 1.

**Table 1:** Basic information of samples.

Variable	Attribute	Number	Percentage
Age	≤25	54	12
	26–30	149	33.1
	31–35	162	36
	36–40	34	7.6
	41–45	18	4
	46–50	21	4.7
	≥51	12	2.7
Sex	Male	266	59.1
	Female	184	40.9
Education level	Senior high school	122	27.1
	Junior college	104	23.1
	Bachelor	118	26.2
	Master	89	19.8
	Doctor	17	3.8

*N* = 450.

### Measurements

The present study adopted measurement scales developed in previous research. Each question item was scored on a 6-point Likert scale measuring the six main variables of narcissistic leadership, hostility toward supervisor, psychological availability, counterproductive work behavior, organizational citizenship behavior and environment uncertainty.

#### Narcissistic leadership (T1)

The single dimensional measurement scale with four items developed by Grafeman et al. (2015) was used. Representative item is “My leader likes to be the center of attention,” the Cronbach’s α is 0.89.

#### Hostility toward supervisor (T2)

The single dimensional measurement scale with six items developed by Watson and Clark (1994) was used. Representative item is “I am full of hostility towards my leader,” the Cronbach’s α is 0.75.

#### Psychological availability (T2)

The single dimensional measurement scale with five items developed by May et al. (2004) was used. Representative item is “I believe I can show proper emotions at work,” the Cronbach’s α 0.76.

#### Counterproductive work behavior (T3)

The single dimensional measurement scale with twenty-three items developed by Yang and Diefendorff (2009) was used. Representative item is “The employee deliberately slows down his work,” the Cronbach’s α is 0.77.

#### Organizational citizenship behavior (T3)

The single dimensional measurement scale with nine items developed by Farh et al. (2007) was used. Representative item is “The employee takes the initiative to help a colleague who has a heavy workload,” the Cronbach’s α is 0.78.

#### Environment uncertainty (T2)

The single dimensional measurement scale with three items developed by De Hoogh et al. (2005) was used. Representative item is “The working environment of our department is full of challenges,” the Cronbach’s α is 0.76.

#### Control variable (T1)

Referring to previous studies (Chen et al., 2022; Wei et al., 2023), this study choose the age, gender, and education level of employees as control variables from common demographic variables. Among them, the age of employees was divided into 7 ranges: 25 years old and below, 26–30 years old, 31–35 years old, 36–40 years old, 41–45 years old, 46–50 years old, 51 years old and above. This approximates a continuous scale. Gender was dummy coded, 0 for male and 2 for female. The education level of employees is divided into high school, college, undergraduate, master’s, doctoral, and it was also treated as a continuous scale.

## Results

### Confirmatory factor analysis

In this research, Confirmatory factor analysis was conducted using Mplus 7.4 on the variables of interest to test the discriminant validity between the variables. The results are shown in Table 2. The six-factor model has the best fitting effect (*χ^2^* = 354.95 df = 237, *χ^2^*/df = 1.50, CFI = 0.97, TLI = 0.96, RMSEA = 0.03, and SRMR = 0.04), indicating that the six variables in this study have good discriminant validity among themselves.

**Table 2:** Confirmatory factor analysis (CFA) results of measurement models.

Model	Factor	*χ^2^*	df	*χ^2^*/df	CFI	TLI	RMSEA	SRMR
Model 1	NL, HTS, PA, CWB, OCB, EU	1665.50	252	6.61	0.61	0.58	0.11	0.09
Model 2	NL, HTS, PA, CWB, OCB, EU	1476.96	251	5.88	0.67	0.63	0.10	0.09
Model 3	NL, HTS, PA, CWB, OCB, EU	987.08	249	3.96	0.80	0.78	0.08	0.07
Model 4	NL, HTS, PA, CWB, OCB, EU	795.46	246	3.23	0.85	0.83	0.07	0.06
Model 5	NL, HTS, PA, CWB, OCB, EU	627.93	242	2.59	0.89	0.88	0.06	0.05
Model 6	NL, HTS, PA, CWB, OCB, EU	354.95	237	1.50	0.97	0.96	0.03	0.04

*N* = 450; NL, Narcissistic Leadership; HTS, Hostility Toward Supervisor; PA, Psychological Availability; CWB, Counterproductive Work Behavior; OCB, Organizational Citizenship Behavior; EU, Environment Uncertainty; +: Two factors combined as one.

Due to the common sources of hostility toward supervisor, psychological availability, environment uncertainty, and narcissistic leadership, in order to avoid common method bias affecting the research results, this study used Harman’s single factor test to test for common method bias. All variables were placed in an exploratory factor analysis to test the non rotated factor analysis results. The results indicate that the variance explained by the first factor is 31.22%, which does not exceed 40% of the cumulative variance, indicating that there is no serious common method bias in the data.

### Descriptive statistics

The means, standard deviations and correlation coefficients of the variables in this study are shown in Table 3. Narcissistic leadership is positively correlated with hostility toward supervisor (*γ* = 0.40, *p* < 0.01) and psychological availability (*γ* = 0.37, *p* < 0.01). Hostility toward supervisor is positively correlated with counterproductive work behavior (*γ* = 0.41, *p* < 0.01). Psychological availability is positively correlated with organizational citizenship behavior (*γ* = 0.42, *p* < 0.01). The results of the above analysis tentatively support the correspondent hypotheses of this study.

**Table 3:** Means, standard deviations and correlations of study variables.

	Mean (M)	Standard deviation (SD)	1	2	3	4	5	6	7	8	9
1. Age (T1)	31.69	6.77									
2. Gender (T1)	0.41	0.49	0.05								
3. Education (T1)	3.53	1.08	0.14**	0.04							
4. Narcissistic leadership (T1)	5.29	0.69	0.03	0.07	−0.02	(0.89)					
5. Hostility toward supervisor (T2)	5.06	0.71	0.11*	−0.06	−0.07	0.40**	(0.75)				
6. Psychological availability (T2)	4.77	0.78	0.08	−0.00	−0.10*	0.37**	0.03	(0.76)			
7. Counterproductive work behavior (T3)	5.33	0.59	0.14**	0.01	0.03	0.36**	0.41**	0.03	(0.77)		
8. Organizational citizenship behavior (T3)	5.32	0.56	0.12**	0.05	−0.03	0.43**	0.06	0.42**	0.06	(0.78)	
9. Environment Uncertainty (T2)	5.40	0.49	−0.08	−0.01	0.02	0.25**	0.21**	0.15**	0.11*	0.19**	(0.76)

*N = 450*. ^*^p < *0.05*. ^**^p < *0.01*. ^***^p < 0.001.

### Main effects test

**Figure 2:**
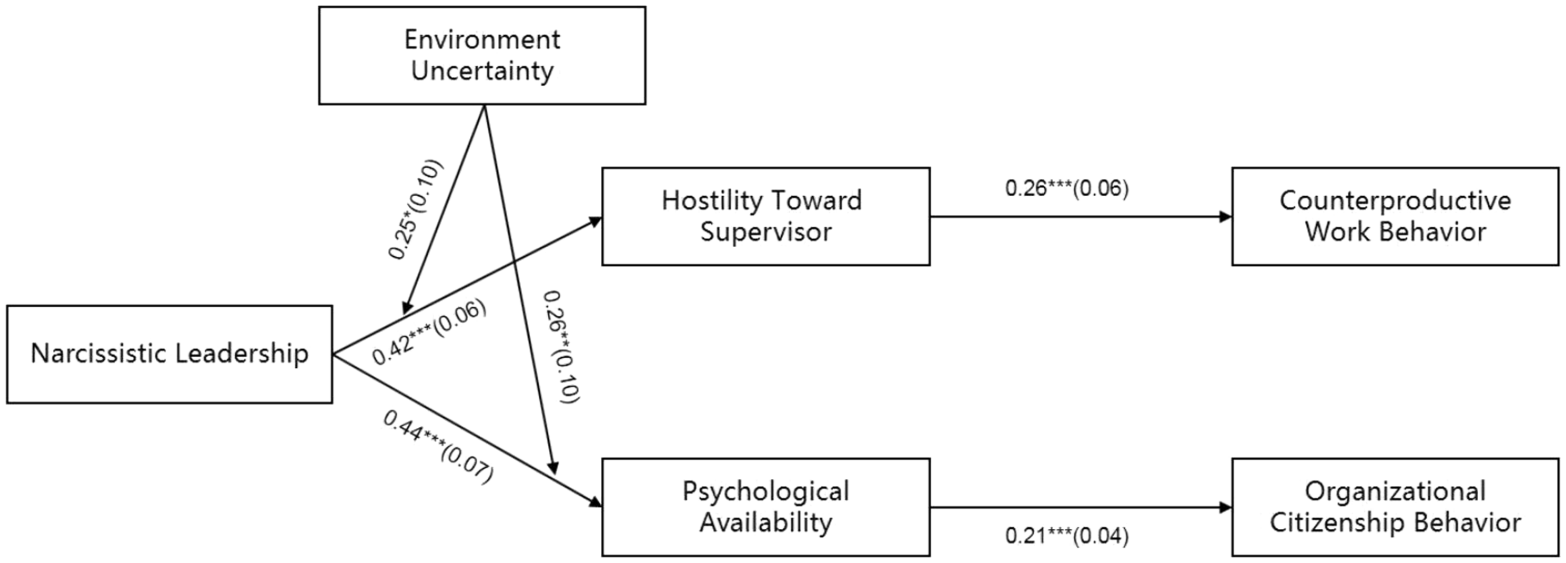
Path coefficients. ^*^p<0.05; ^**^p < 0.01; ^***^p < 0.001; Coefficients in the graph are standardized coefficients with standard errors in parentheses; control variables are age, gender and education background.

In this study, Mplus 7.4 was used to test the fit indicators and related hypotheses of the structural equation model. Firstly, according to the fitting index of the theoretical model (*χ^2^* = 360.30, df = 241, *χ^2^*/df = 1.50, CFI = 0.97, TLI = 0.96, RMSEA = 0.03, SRMR = 0.04), it can be judged that the model has a good fit. Secondly, the results of the path analysis are shown in Figure 2, where narcissistic leadership is positively correlated to hostility toward supervisor (*β* = 0.42, *p* < 0.001) and psychological availability (*β* = 0.44, *p* < 0.01), so H1, H2 are verified.

### Mediation effects test

In this study, Bootstrap (repeated sampling 5,000 times) was used to test the mediation effects of hostility toward supervisor and psychological availability, and the results are shown in Table 4. The mediation effect of hostility toward supervisor (*β* = 0.11, *p* < 0.001) is significant and the 95% confidence interval is [0.059, 0.169], excluding 0, so H3 is verified. The mediation effect of psychological availability (*β* = 0.09, *p* < 0.001) is significant and the 95% confidence interval is [0.056, 0.130], excluding 0, so H4 is verified.

**Table 4:** The mediating effect of hostility toward supervisor and psychological availability.

Indirect path	Indirect effect *β*	95% confidence interval CI
Path 1: NL → HTS → CWB	0.11***	(0.059, 0.169)
Path 1: NL → PA → OCB	0.09***	(0.056, 0.130)

*N* = 450; NL, Narcissistic Leadership; HTS, Hostility Toward Supervisor; PA, Psychological Availability; CWB, Counterproductive Work Behavior; OCB, Organizational Citizenship Behavior; Bootstrap = 5000times.

### Moderation effect test

**Figure 3:**
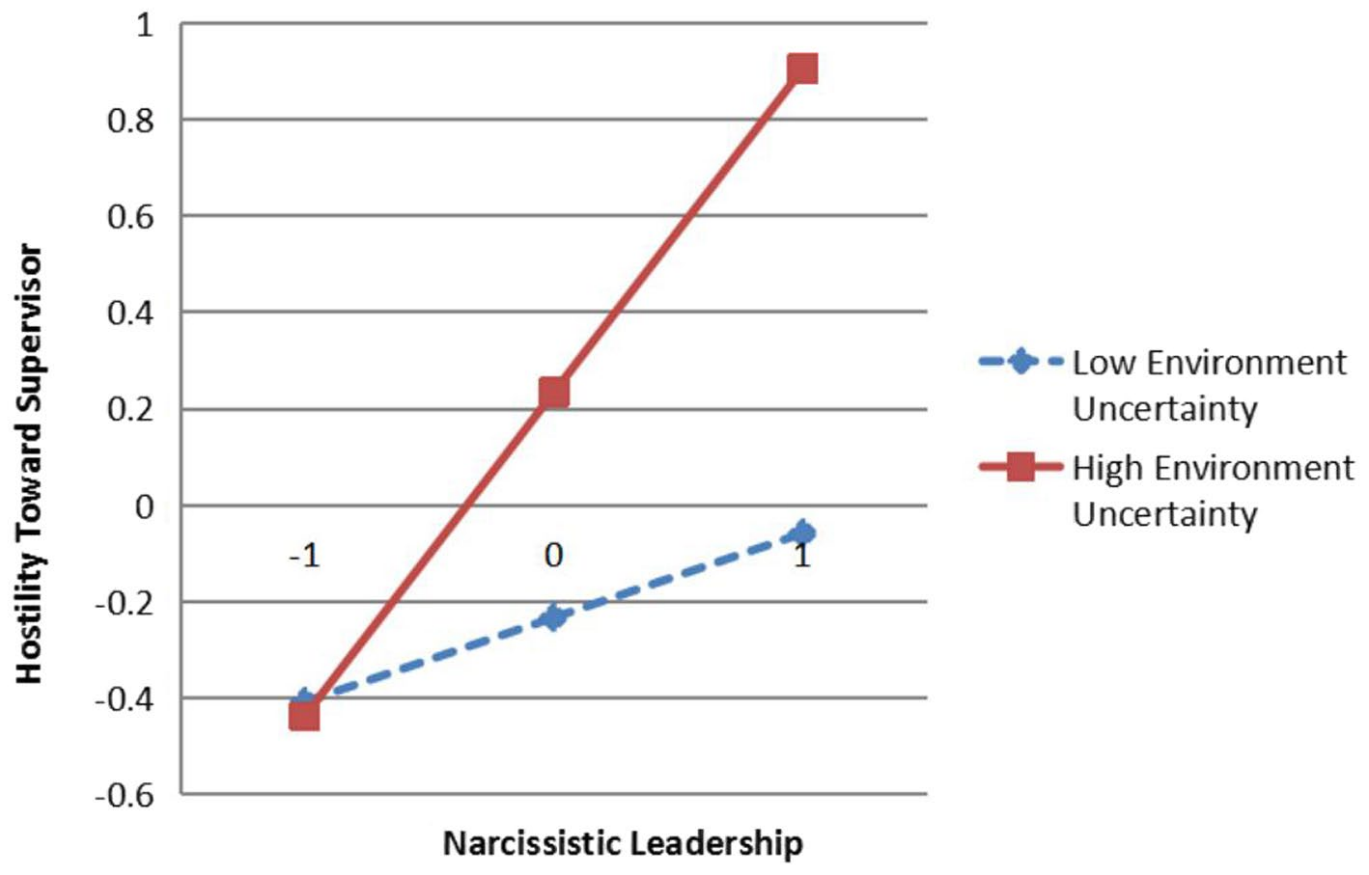
The Moderating effects of environmental uncertainty on narcissistic leadership and hostility toward supervisor (a). *N* = 450; NL, Narcissistic Leadership; EU, Environment Uncertainty.

**Figure 4:**
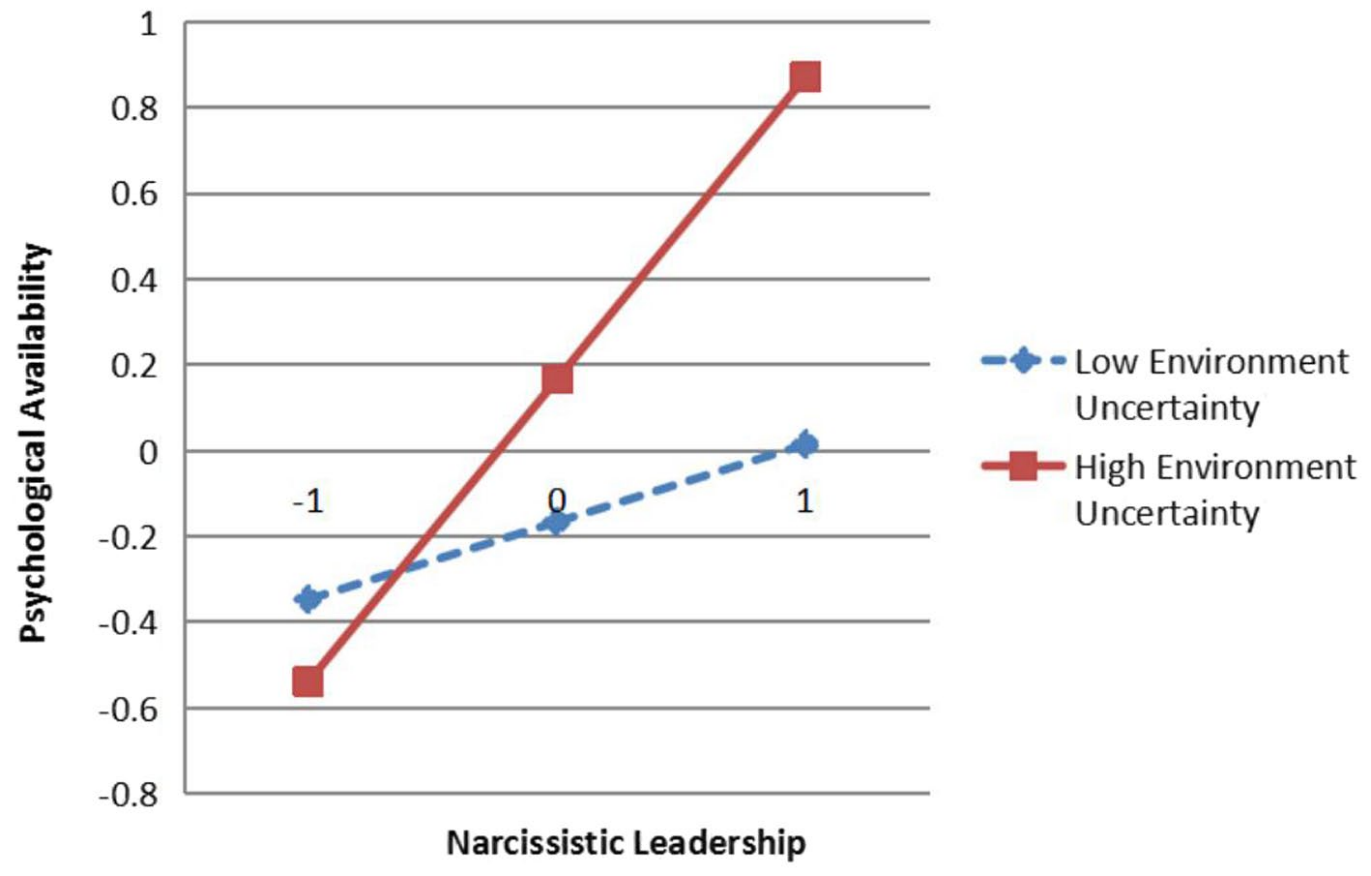
The Moderating effects of environmental uncertainty on narcissistic leadership and psychological availability (b). *N* = 450; NL, Narcissistic Leadership; EU, Environment Uncertainty.

The interaction term between narcissistic leadership and environmental uncertainty has a significant effect on hostility toward supervisor (*β* = 0.25, *p* < 0.05), and psychological availability (*β* = 0.26, *p* < 0.01), indicating that environmental uncertainty significantly moderates the relationship between narcissistic leadership and hostility toward supervisor as well as the relationship between narcissistic leadership and psychological availability. To further explain the moderation effect of environmental uncertainty, a simple slope test was conducted as suggested by Aiken and West (1991) and plotted as shown in Figures 3, 4.

The results indicate that when employees perceive lower levels of environmental uncertainty, the positive effects of narcissistic leadership on hostility toward supervisor (*β* = 0.30, *t* = 5.89, *p* < 0.001) and psychological availability (*β* = 0.31, *t* = 5.49, *p* < 0.001) are weaker; when employees perceive higher levels of environmental uncertainty, the positive effects of narcissistic leadership on hostility toward supervisor (*β* = 0.55, *t* = 8.25, *p* < 0.001), and psychological availability (*β* = 0.57, *t* = 7.72, *p* < 0.001) are stronger. That is, the higher the level of employees’ perceived environmental uncertainty is, the stronger the positive effects of narcissistic leadership on hostility toward supervisor and psychological availability are, so H5 and H6 are verified.

To examine the moderation effect of environmental uncertainty, this study used Bootstrap (repeated sampling 5,000 times) to test the moderated mediation effect. The results are shown in Table 5, where the mediation effect of hostility toward supervisor between narcissistic leadership and counterproductive work behavior is moderated by environmental uncertainty, i.e., for employees who perceive higher levels of environmental uncertainty (one standard deviation above the mean), the indirect effect of narcissistic leadership on counterproductive work behavior via hostility toward supervisor is significantly higher than for employees who perceive lower levels of environmental uncertainty (one standard deviation below the mean) and the difference is significant (*β* = 0.06, *p* < 0.05) and the 95% confidence interval is [0.011, 0.129], excluding 0, so H7 is verified.

**Table 5:** Test results for moderated mediated effects.

EU	NL → HTS → CWB		NL → PA → OCB	
	Indirect effect *β*	95% confidence interval CI	Indirect effect *β*	95% confidence interval CI
Low EU	0.08**	(0.036, 0.141)	0.07***	(0.039, 0.100)
High EU	0.14***	(0.076, 0.223)	0.12***	(0.077, 0.189)
Discrepancy	0.06*	(0.011, 0.129)	0.05*	(0.014, 0.104)

*N* = 450; NL, Narcissistic Leadership; HTS, Hostility Toward Supervisor; PA, Psychological Availability; CWB, Counterproductive Work Behavior; OCB, Organizational Citizenship Behavior; EU, Environment Uncertainty; Bootstrap = 5,000 times.

The mediation effect of psychological availability between narcissistic leadership and organizational citizenship behavior is moderated by environmental uncertainty, i.e., for employees who perceive higher levels of environmental uncertainty (one standard deviation above the mean standard deviation), the indirect effect of narcissistic leadership on organizational citizenship behavior via psychological availability is significantly higher than for employees who perceive lower levels of environmental uncertainty (one standard deviation below the mean), and the difference is significant (*β* = 0.05, *p* < 0.01) and the 95% confidence interval is [0.014, 0.104], excluding 0, so H8 is verified.

## Discussion

### Theoretical implications

First, this study expands the outcome variables and research perspectives of narcissistic leadership. Previous studies have mostly focused on exploring the negative effects of narcissistic leadership (Carnevale et al., 2018; Badar et al., 2023). Although a few scholars have explored the positive effects of narcissistic leadership (Owens et al., 2015), due to their single research perspective (Wang et al., 2022), it is not conducive to a systematic understanding of the role played by narcissistic leadership. This paper chooses two opposing extra-role behaviors, counterproductive work behavior and organizational citizenship behavior, verifying narcissistic leadership’ double-edged sword effect on employees, and to some extent addressing the controversy of existing studies on the effectiveness of narcissistic leadership (Nevicka et al., 2011b), providing a theoretical foundation and empirical support for narcissistic leadership research.

Second, this study reveals the influence mechanism of narcissistic leadership from the perspective of resource gains and losses. The results show that the “dark side” of narcissistic leadership not only triggers hostility toward supervisors but also promotes psychological availability, which is helpful for scholars to understand the effectiveness of narcissistic leadership more comprehensively. In addition, the text expands on the traditional single-mediator study of narcissistic leadership (Resick et al., 2009; Owens et al., 2015) by exploring the mechanisms of influence between narcissistic leadership and employees’ work behavior from both affective and perceptual paths.

Finally, this study incorporates environmental uncertainty as a contextual factor into the research framework (Waldman et al., 2001). This paper finds that employees experience different external environmental pressures in contexts with different levels of environmental uncertainty (Zhao et al., 2021), which makes employees differ in their sensitivity to leadership behaviors and ultimately leads to differences in the effects of narcissistic leadership on their hostile emotions and psychological availability. Therefore, this paper enriches the study of environmental uncertainty to a certain extent and provides new research ideas for future research on the relationship between leadership behaviors and employees’ emotion, cognition and behavioral responses.

### Practical implications

First, we suggest that organizations should take a dialectical view of narcissistic leadership and try to suppress its “dark” side as much as possible. The findings of this paper suggest that narcissistic leadership has both positive and constructive aspects, as well as negative and destructive aspects in the context of today’s dynamic, complex and ever-changing society (Neeley and Leonardi, 2022). Therefore, in practice, it is important to distinguish between the positive and negative effects of narcissistic leadership, and to give full play to its positive effects and reduce its negative ones. Specifically, the organization should establish sound rewards and punishment measures in practice, and give corresponding punishment to leaders who only focus on individual interests and ignore collective interests, so as to prevent narcissistic leaders from putting personal interests above collective interests (Badar et al., 2023). At the same time, accountability should be established in the organization to clarify the responsibilities of leaders and improve the transparency of leadership behavior, and enterprises should implement a democratic voting system for major decisions to inhibit narcissistic leaders from taking risks with collective wisdom. Finally, the organization should create a culture of mutual help and love to reduce the indifference and insensitivity of narcissistic leaders and increase the motivation and satisfaction of subordinates (Schyns et al., 2023).

Second, we suggest that organizations should pay attention to employees’ emotions and their physical, emotional, cognitive care as well as guidance. On the one hand, managers should recognize that employee hostility may be potentially harmful to organizations. Especially in the process of organizational digital transformation (Neumeyer and Liu, 2021), inducing hostile emotions among employees may lead to counterproductive work behaviors, which not only affects the organizational climate but also jeopardizes organizational performance. Therefore, organizations should face up to the harmful effects of such negative emotions. Leaders should focus on observing the emotional changes of their subordinates in their daily work and lead them in an open and tolerant way, create a relaxed and comfortable working environment for employees, and give them the necessary psychological counseling to minimize the negative effects of employees’ hostile emotions and encourage them to complete their work with a positive mental outlook (Siebens, 2019). On the other hand, emotional concern from leaders, encouragement at work, timely and effective feedback and recognition, and good HR practices can increase employees’ perceived resource availability and promote organizational citizenship behaviors. Therefore, organizations should create a seamless communication channel between the top and bottom, and open up avenues for subordinates to give feedback on the behavior of their supervisors, such as opening an internal company community and message wall and other corresponding platforms to prompt leaders to respond positively to the needs of employees and enrich the availability of resources for employees (Cai et al., 2018).

Third, we suggest that organizations should maintain the necessary attention to the external environment. Organizations can maintain continuous attention to the changes of the external environment, so the sustainable development of organizations can be maintained at a certain height, and the vitality and competitiveness of organizations can be ensured. Therefore, organizations must hold an open attitude, especially in today’s digital age full of crises (Solberg et al., 2020). The internal and external processes of organizations are equally important. Only by combining internal and external learning can it provide enough valuable resources and information for the development of organizations. Leaders should consider the impact of the external environment on the organizational process. When the external environment is highly uncertain, the effectiveness of leadership behavior will be limited to a certain extent (Zhao et al., 2021). Therefore, in real life, we should correctly avoid the negative impact of the external environment on the development of organizations, obtain development opportunities, and avoid threats.

### Limitations and directions for future research

There are some limitations and shortcomings in this paper, which need to be improved in subsequent studies. First, this paper used a cross-sectional research design, which may not accurately reveal the dynamic processes among variables, and the persuasiveness of the findings can be improved in the future through methods such as experience sampling method and logbook method.

Second, the scale of narcissistic leadership used in this paper is a unidimensional scale, and the three-dimensional scale developed by Wink (1992) can be selected for further analysis of the double-edged sword effect of narcissistic leadership.

Third, the control variables included in this paper were limited, and other control variables that may influence the findings, such as time spent with superiors, can be included in the future study.

Fourth, considering the context of interpersonal relationships in China, hostility toward supervisor as a mediation variable may appear more direct in emotional expression. In the future, other mechanisms can be further introduced or unexplored mediation variables (such as flattery, silence, etc.) can be further explored from other perspectives, and differences in their mechanisms of action can be compared based on our research results.

Fifth, this paper explored the mechanism of narcissistic leadership and employee behavior at the individual level, and future studies may attempt to measure the variables at the team level to further validate the effect of narcissistic leadership on employee behavior.

Finally, the selection of outcome variables in this study appears relatively ordinary. In future research, we can choose variables that are more closely related and novel to the current era background as the results for further discussion, such as knowledge hiding behavior and knowledge sharing behavior, job burnout and work activity.

## Conclusion

In the past, many studies have emphasized the drawbacks of narcissistic leadership, especially its negative impact on employee behavior (Schyns et al., 2023). Although some studies have found positive mechanisms of narcissistic leadership (O'Reilly et al., 2014), we still know little about whether narcissistic leadership can lead to two completely opposite work behaviors among employees at the same time. It is worth noting that the effectiveness of narcissistic leadership is likely to be influenced by the gains and losses of employee resources. Therefore, when exploring this issue, we established and tested the impact of narcissistic leadership on employee counterproductive behavior and organizational citizenship behavior.

As predicted, we find that narcissistic leadership can increase employees’ hostile emotions, thereby inducing them to engage in unproductive behavior. At the same time, the psychological accessibility of employees will also be improved, which will promote the occurrence of organizational citizenship behavior. Throughout this entire process, environment uncertainty plays a promoting role. We hope that this study can draw the attention of scholars to pay attention to the double-edged sword effect of leader’s narcissistic behavior on employees, and call on leaders to pay attention to providing sufficient resource support for employees to help them better adapt to narcissistic leadership styles in order to avoid negative work performance. In addition, this study also provides strong research basis for further exploring narcissistic leadership in the future.

## Data availability statement

The datasets presented in this study can be found in online repositories. The names of the repository/repositories and accession number(s) can be found in the article/supplementary material.

## Ethics statement

Ethical review and approval was not required for the study on human participants in accordance with the local legislation and institutional requirements. Written informed consent from the patients/participants or patients/participants legal guardian/next of kin was not required to participate in this study in accordance with the national legislation and the institutional requirements.

## Author contributions

HC: Data curation, Investigation, Project administration, Visualization, Writing – original draft, Writing – review & editing. LZ: Methodology, Formal analysis, Funding acquisition, Writing – review & editing. LW: Investigation, Resources, Writing – review & editing. JB: Investigation, Resources, Formal analysis, Methodology, Writing – review & editing. ZZ: Formal analysis, Methodology, Writing – review & editing.
